# Oncolytic Viruses in Combination Therapeutic Approaches with Epigenetic Modulators: Past, Present, and Future Perspectives

**DOI:** 10.3390/cancers13112761

**Published:** 2021-06-02

**Authors:** Annalisa Chianese, Biagio Santella, Annalisa Ambrosino, Debora Stelitano, Luca Rinaldi, Massimiliano Galdiero, Carla Zannella, Gianluigi Franci

**Affiliations:** 1 Department of Experimental Medicine, University of Campania “Luigi Vanvitelli”, 80138 Naples, Italy; annalisa.chianese@unicampania.it (A.C.); annalisa.ambrosino@unicampania.it (A.A.); debora.stelitano@unicampania.it (D.S.); massimiliano.galdiero@unicampania.it (M.G.); 2 Section of Microbiology and Virology, University Hospital “Luigi Vanvitelli”, 80138 Naples, Italy; biagio.santella@studenti.unicampania.it; 3 Department of Advanced Medical and Surgical Sciences, University of Campania “Luigi Vanvitelli”, 80138 Naples, Italy; luca.rinaldi@unicampania.it; 4 Department of Medicine, Surgery and Dentistry “Scuola Medica Salernitana”, University of Salerno, 84081 Baronissi, Italy

**Keywords:** oncolytic virus, combination treatment, cancer, epigenetic, tumor resistance, HCC, DNA methyltransferase, histone deacetylases, microRNA

## Abstract

**Simple Summary:**

Cancer rates have been accelerating significantly in recent years. Despite notable advances having been made in cancer therapy, and numerous studies being currently conducted in clinical trials, research is always looking for new treatment. Novel and promising anticancer therapies comprise combinations of oncolytic viruses and epigenetic modulators, including chromatin modifiers, such as DNA methyltransferase and histone deacetylases, and microRNA. Combinatorial treatments have several advantages: they enhance viral entry, replication, and spread between proximal cells and, moreover, they strengthen the immune response. In this review we summarize the main combination of therapeutic approaches, giving an insight into past, present, and future perspectives.

**Abstract:**

According to the World Cancer Report, cancer rates have been increased by 50% with 15 million new cases in the year 2020. Hepatocellular carcinoma (HCC) is the only one of the most common tumors to cause a huge increase in mortality with a survival rate between 40% and 70% at 5 years, due to the high relapse and limitations associated with current therapies. Despite great progress in medicine, oncological research is always looking for new therapies: different technologies have been evaluated in clinical trials and others have been already used in clinics. Among them, oncolytic virotherapy represents a therapeutic option with a widespread possibility of approaches and applications. Oncolytic viruses are naturally occurring, or are engineered, viruses characterized by the unique features of preferentially infecting, replicating, and lysing malignant tumor cells, as well as activating the immune response. The combination of oncolytic virotherapy and chemical drugs are arousing great interest in the tumor treatment. In this scenario, novel and promising anticancer therapies comprise combinations of oncolytic viruses and epigenetic modulators or inhibitors of the signalling pathways. Combination treatments are required to improve the immune response and allow viral entry, replication, and diffusion between proximal cells. In this review, we summarize all combination therapies associated with virotherapy, including co-administered inhibitors of chromatin modifiers (combination strategies) and inserted target sites for miRNAs (recombination or arming strategies).

## 1. Introduction

Cancer is a well-known serious health issue associated with high morbidity and mortality, second only to cardiovascular diseases in the world [1]. The World Health Organization (WHO) declared that over 18.1 million cases of cancer were diagnosed and 9.6 million deaths occurred in 2018 [2], with an estimated increase of 60% by 2040 due to ageing and increasing of the world’s population [3]. Among tumors, liver cancer is the one associated with the highest death rate [2,4] and whose onset is increasing every year [5,6,7]. The most frequent liver cancer is hepatocellular carcinoma (HCC), commonly associated with chronic hepatitis B virus (HBV) and hepatitis C virus (HCV) infections, alcohol abuse, metabolic liver disease, smoking, and obesity [8,9,10,11]. Therapeutic treatments are very complicated, as only a few patients, especially in the initial phase, can undergo surgery. Conversely, oral therapy with sorafenib, a kinase inhibitor, is indicated for patients in more advanced stages of cancer, but, unfortunately, is frequently associated with the onset of resistance within six months of treatment [12,13,14,15,16,17]. Furthermore, alongside this problem, long-term use of most chemotherapy drugs, including sorafenib, results in toxicity and ineffectiveness of the drug [18,19,20,21]. Altogether, these issues push toward the research of alternative treatments. For the treatment of resistant and orphan tumors, oncolytic virotherapy represents a real opportunity. Virotherapy is a new promising approach against different types of cancers through the use of oncolytic viruses (OVs). They are naturally occurring, or genetically modified, viruses able to infect, replicate, and lyse several malignant tumor cells. Virotherapy was born in the 19th century [22,23,24], and in the 1950s–1970s, the first clinical trials began and live viruses were deliberately injected into patients with cancer to promote tumor regression [22]. In the last decade, thanks to genetic engineering and the advent of in vitro experiments, the viral genome has been easily manipulated and modified to make viruses more selective for cancer cells and minimize their potential side effects [25,26,27], causing a great burst of oncolytic virotherapy. OVs act through a dual-mode: on the one hand, they are able to lyse the tumor cells and, on the other, they can influence the immune response. Regarding their effect on the immune response, OVs are able to regulate it in a fine way: (i) on the one hand, they escape by inhibiting the production of cytokines/chemokines from infected cells or by blocking apoptosis from cytotoxic T lymphocytes [28,29,30]; (ii) on the contrary, they can alter the immunosuppressive state in which the tumor microenvironment is, stimulating the immune response and the antitumor effect, so that the recruited immune cells recognize the tumor and lead to its destruction. These benefits have led to a strong increase of clinical trials. Indeed, actually we recorded almost 150 trials in progress and over 70 potential OVs that are ready to be introduced in the therapeutic treatment against different types of cancer [31,32,33,34,35,36,37,38]. Different OVs have been studied and manipulated for cancer therapy, and among them there are Adenovirus (AdV) [39,40,41,42,43], Herpes Simplex Virus (HSV) [44,45,46,47,48], Coxsackie virus [49,50,51], Reovirus [52,53,54,55], Measles [56,57,58,59], Parvovirus [60,61], Vesicular Stomatitis Virus (VSV) [62,63,64], and Newcastle Disease Virus (NDV) [26,65,66]. Since 2018, only three OVs have been approved for cancer therapy. ECHO-7 Rigvir virus strain, approved for the treatment of melanoma in Latvia, and other two genetically modified OVs, the AdV H101 (Oncorine) for the treatment of head, neck, and esophagus squamous cell carcinoma in China, and, lastly, the HSV type 1 (HSV-1) Imlygic which was recently approved in the United States, Australia, and Europe for the treatment of melanoma. To date, there are a plethora of oncolytic viruses used in clinical trials reported in Table 1. 

The replicative cycles and structural characteristics of these OVs differ drastically, but they share a common and contrasting mechanism of action. They are able to kill tumor cells without damaging healthy tissues. In fact, cancer cells have peculiar characteristics able to strengthen viral replication [124]: (i) they oppose apoptosis causing indefinite proliferation [125]; (ii) they are lacking in cellular antiviral response pathways [126,127]; (iii) they create a hypoxic environment in which OVs can replicate [128,129,130]; (iv) they usually show an over-active RAS signalling pathway, which allows viruses such as reovirus and vaccinia virus to specifically replicate and lyse tumor cells, in which the PKR pathway is not active. On the contrary, in normal healthy cells, the PKR pathway is on, and the production of viral particles, and therefore viral replication, are inhibited [131]; and (v) cancer cells can also expose on their surface viral entry receptors, e.g., nectin and herpesvirus entry mediator (HVEM), used by HSV, which are overexpressed in melanoma and different carcinoma [132]. As a result, OVs replicate and lyse selectively tumor cells spreading viral progeny and other products of oncolysis. The release of infectious viral progeny allows oncolysis amplification also towards neighboring tumor cells. Cytokines, such as the tumor necrosis factor-α (TNFα), interferon γ (IFNγ), and interleukin-12 (IL-12), are also released and induce the maturation of antigen-presenting cells (APCs), which in turn activate natural killer (NK) cells, CD4 + and CD8 + T cells. The cytotoxic effect induced by these cells is essential in determining tumor regression even in distant sites, not directly exposed to the virus. However, contrary to this pattern of action, OVs can also trigger an antiviral immune response of the host capable of neutralizing the virucidal action by antiviral antibodies. At the same time, NKs can also block the action of viruses by killing infected cells. The balance between the immune-mediated viral clearance and the induction of antitumor immunity is very delicate and depends mainly on the characteristics of the virus and the tumor microenvironment [133]. Nevertheless, there are still many drawbacks to overcome in order to achieve the full effectiveness of virotherapy. In this scenario, the main limitations of virotherapy are represented by off-target effects which can occur following viral infection. Potential adverse phenomena, such as viral encephalitis caused by HSV [134], require risk monitoring, which must be considered before treating patients. Infectious viral progeny is released following cancer cells’ lysis and it could cause secondary peaks of viremia and thus adverse effects, regardless of the viral dose used in the treatment. Regardless of OV effectiveness, (i) the host’s antiviral immune responses [133], (ii) the tumor microenvironment [135], (iii) the size of the viral inoculum, and (iv) the treatment time represent a barrier against the success of virotherapy [136]. Solving these problems can lead to considering virotherapy as an elite treatment in cancer care. Considering that the risk-benefit ratio of infectious agents will inevitably accompany OV-based therapies, it is obvious that the transition into clinics requires a significant improvement in safety profiles, as the potential side effects caused by the injection of live viruses and their probable mutation towards a more pathogenic phenotype remain a serious issue. In general, single-agent therapies are poorly efficient in cancer care treatment. In order to increase efficacy, recently synergy between OVs and chemotherapy or radiotherapy has been tested. Combination treatments, particularly with known chemotherapy agents, have shown an increase in sensitivity, with significantly improved results for several oncolytic viruses [137]. The challenge will be to identify the most efficient combinations with OVs for cancer therapy, especially the most functional ones in the treatment of tumors resistant to other therapies, i.e., HCC. Novel and promising anticancer therapies involve combinations of OVs and other chemotherapy drugs, i.e., epigenetic modulators or inhibitors of the signalling pathway [138]. The targets include both chromatin modifiers, such as DNA methyltransferase (DNMT) and histone deacetylases (HDAC), and microRNA (miRNA). It has been already reported that some viral infections can be strongly regulated by epigenetic modulators. For example, strong evidence demonstrated that HCC can also persist after clearance because of epigenetic changes induced by HCV [139].

Combinatorial treatments are required to improve the immune response and allow viral entry, replication, and diffusion between adjacent cells. In this review we discuss firstly the major viral families used in virotherapy and the clinical trials in which OVs are used; then we focus on the specific combinatorial therapies, including co-administered inhibitors of chromatin modifiers (combination strategies) and inserted target sites for miRNAs (recombination or arming strategies).

## 2. Viral Families Most Used in Virotherapy

### 2.1. Herpesviridae

*Herpesviridae* is a large family with about 100 viruses causing disease in many animals, including humans, monkeys, fish, and birds [140,141]. HSV is a virus consisting of a 150 kb double-stranded DNA genome characterized by repeated sequences, direct or inverted, which delimit unique long (UL) and short regions (US) ensuring its replication and recombination. It is wrapped in an icosahedral capsid consisting of 162 capsomers, in turn separated from the envelope by the tegument, which contains enzymes necessary for the viral replication [142,143,144]. Imlygic, also called talimogene laherparepvec (T-Vec) was in 2015, the first OV to be approved in the United States for the treatment of melanoma, and in Europe and Australia in 2016 [145]. T-Vec is a genetically modified HSV-1 with a deletion in γ-34.5 and α-47 genes and addition of two copies of the gene for the Colony Stimulating Factor of human Granulocyte-Macrophages (GM-CSF) [146]. Many ongoing clinical trials evaluate the efficacy of T-VEC alone or in combination with various other therapies [147,148,149,150,151], and its safety profile has been investigated in patients with various metastatic cancers, including breast, gastrointestinal, and melanoma [148,152,153]. In all, intralesional administration of T-Vec was well tolerated, without inducing strong symptoms and stimulating strong anti-cancer effects [154]. In phase II studies, the immune response rate was evaluated, reporting an increase in CD8+ T cells and a decrease in CD4+, detected in biopsy samples from regressive lesions [155]. Robert et al. compared the effects of T-VEC and GM-CSF alone in patients with unstabilized melanoma [156]. The results with T-VEC indicated a higher DRR (an objective response that lasted continuously ≥6 months), with an increase in survival and overall response rate compared to GM-CSF alone. Globally, these effects support T-VEC as good oncolytic immunotherapy against melanoma, which not only suppresses tumor growth, but improves the systemic anti-tumor immunity, as reported in a phase III of clinical trial [157]. The oncolytic triple mutant G47Δ was created by removing the α47 gene from G207, a parental second-generation oHSV-1 characterized by deletions in both copies of the γ34.5 gene and a β-galactosidase (LacZ) insertion [157,158]. In 2017, Daiichi Sankyo Company designated G47Δ as an orphan drug for the treatment of malignant glioma [159].

R849 is an oncolytic strain of HSV-1 containing a LacZ gene inserted in the place of γ-34.5, responsible for HSV-1 neurovirulence. Once viral infection begins, there is a translocation of p65, a component of NF-kB into the nucleus. The OV rQNestin34.5, a HSV-1 mutant expressing ICP34.5 under the control of the Nestin promoter, was found to be useful in the treatment of glioma both in vitro and in vivo [160].

### 2.2. Adenoviridae

AdVs are naked viruses consisting of a linear double-stranded DNA of approximately 35 kb that encodes over 40 proteins [161]. The genome is enclosed in the capsid, consisting of 240 capsomers with an icosahedral structure. Pathogenic AdVs for humans are represented by AdV C, AdV C5, and AdV C2 [162]. H101 (Oncorine) is a genetically modified adenovirus carrying a deletion of the E1B gene and four deletions in viral E3, and it is characterized by an alternative selectivity towards p53 positive tumors [163,164]. H101 was approved in China in 2005 for the treatment of head, neck, and esophageal cancer [165]. 

ONYX-015 is a modified AdV very similar to H101 consisting of a deletion of the E1B-55K and E3 genes. Several clinical studies have demonstrated that, when administered inside the tumor, ONYX-015 is effective without causing side effects [166,167], showing an anticancer effect also in patients with hepatobiliary tumors [168]. Phase II trial reported the results of standard or hyper fractionated treatment with ONYX-015, lasting 21 days, in patients with refractory head and neck carcinoma. It underlined a modest anti-tumor activity with mild fever and pain at the injection site, confirming safety and low toxicity [169]. Important results have been obtained following the combined treatment of ONYX-015 with cisplatin and 5-fluorouracil in patients with head and neck cancer. The absence of tumor progression was observed compared to tumors treated with chemotherapy alone, which, on the contrary, had progressed [170]. Unfortunately, the high serum prevalence in vaccines against numerous adenovirus serotypes, limited intravenous administration in the treatment of metastatic tumors [171]. However, there are ongoing studies to overcome this obstacle, with the use of adenoviral vectors with modified proteins. DNX-2401 (tasadenoturev) or Delta-24-RGD, is an oncolytic adenovirus used in the treatment of brain tumors, in particular glioma and glioblastoma [172]. It is a virus with a 24 bp deletion in the E1A gene and an RGD motif in the fiber protein. In glioma tumor cells, the Rb pathways are altered, so the deletion in the E1A gene causes the virus to replicate independently of its binding to Rb; while the target of RGD is represented by integrins (avb3 and avb5), present on the surface of tumor cells. These mutations facilitate selective replication only in Rb-dysfunctional tumor cells [173].

As reported in the literature in a study conducted by Lang et al., treatment with tasadenoturev resulted in increased survival of patients with glioma [174].

Another oncolytic adenovirus used for the treatment of Rb-dysfunctional tumors is VCN-01, also characterized by the Delta24-E1A deletion. It has been shown that the single use of the virus determines tumor necrosis and an improvement of survival in ocular tumors [172]. Clinical trials of VCN-01 in combination with other drugs (gemcitabine and Abraxane^®^) are ongoing in phase II, for the treatment of pancreatic adenocarcinoma. To date, H101 remains the only adenovirus approved for cancer therapy, though it has been used only in combination with chemotherapy.

### 2.3. Rhabdoviridae

VSV belongs to the *Rhabdoviridae* family: it is an enveloped virus consisting of a negative single-stranded RNA genome. It is approximately 11 kb in length and encodes five genes that synthesize nucleoprotein (N), phosphatase protein (P), matrix protein (M), glycoprotein (G), and the large polymer subunit protein (L) [175]. VSVΔM51 is an oncolytic virus carrying a deletion in the M gene, and it causes cell death in tumors by activating the apoptotic pathway [176]. However, VSVΔM51 has never been used in any clinical trials.

### 2.4. Reoviridae

*Reoviridae* are naked viruses with double-stranded segmented RNA of approximately 10–12 kb. The genome is surrounded by an icosahedral capsid and the segments encode eight structural and four non-structural proteins [177]. Reolysin, also called Pelareorep, represents an OV developed by Oncolytics Biotech. It is an unmodified reovirus extensively evaluated in preclinical models and clinical studies [178]. It preferentially replicates and lyses cancer cells [138], then the infected tumor cells release cytokines, activating immune cells and leading to cancer cell death [179]. Currently, Reolysin has been extensively evaluated in several clinical trials [105,180,181,182,183]. 

### 2.5. Parvoviridae

Parvoviruses are small viruses belonging to the *Parvoviridae* family. They consist of a linear single-strand DNA of approximately 5 kb, enclosed in an icosahedral capsid, which is in turn made up of structural proteins, VP-l and VP-2 [144]. H-1PV is a naturally occurred OV characterized by NS1 and NS2 proteins, whose expression is under the control of the early promoter P4 [184,185,186]. NS1 protein is responsible for cytotoxicity, and it can cause apoptosis, cell lysis, and accumulation of reactive oxygen species (ROS) in cells [187]. In particular the study of Josupeit R. et al. [188] showed, for the first time, the ability of high-grade glioma stem cells to lytic infection lead by Parvovirus H-1, not so far reported for any oncolytic virus. H-1PV was able to prevent adult glioma stem-like cells from subcutaneous engraftment in immunodeficient mice and the tumorigenicity of glioblastoma multiforme. In a first phase I/IIa conducted by Geletneky K. et al. [60] (ParvOryx), the oncolytic H-1 Parvovirus did not show signs of systemic inflammation, excessive immune activation, or main organ toxicity in the patient cohort. Only one patient showed a progressively deteriorating level of consciousness two days after intracerebral administration of ParvOryx. Moreover, tumors from six ParvOryx-treated patients showed strong leukocytic infiltration and, finally, the capacity for establishing an immunogenic tumor microenvironment.

### 2.6. Paramyxoviridae

Parainfluenza viruses are human pathogens belonging to the *Paramyxoviridae* family. The most outstanding for humans are mumps virus, measles virus, respiratory syncytial virus (RSV), and human parainfluenza viruses (HPIV), divided into HPIV-1, HPIV-2, HPIV-3, and HPIV-4 [189]. All are enveloped viruses and have a non-segmented single-stranded RNA genome with a negative polarity of about 15-19 kb. The genome is enclosed by a helical nucleocapsid, which is associated with the matrix protein (M) covering the envelope internally. The envelope contains two glycoproteins, hemagglutinin/neuraminidase (HN), or hemagglutinin (H) or G, which acts as an anti-receptor, and fusion glycoprotein (F), which promotes interaction with the host cell [190]. Inside the envelope, the helical nucleocapsid core contains the RNA genome and the nucleocapsid (NP), phosphoprotein (P), and large (L) proteins, which initiate intracellular virus replication. Parainfluenza virus 5 (PIV5), also known as Simian 5 (SV5), has the same characteristics previously mentioned, and, in addition, it encodes the typical structural small integral membrane protein (SH). P and cysteine-rich protein (V) share 164 amino acids [191]. The oncolytic mutant P/V-CPI was created by substituting different amino acids in the P/V region of PIV5. The use of this mutant, in association with the HDACi scriptaid, enhanced apoptosis of small cell lung cancer, and laryngeal carcinoma cells [192,193]. Measles, mumps, and NDV are considered oncolytic vectors as they are able to activate the immune response and have cytopathic properties [192].

### 2.7. Poxviridae

Vaccinia virus (VV) belongs to the *Poxviridae* family. It is an enveloped virus consisting of 130–250 kbp double-stranded linear DNA, and its replication occurs within the cytoplasm. Several studies showed the presence of different VV mutants with deletions in different genes, including thymidine kinase (TK) [137]. TG6002 is a TK and ribonucleotide reductase (RR) deleted OV; it has immunomodulating and anticancer properties since it codes for the cytosine deaminase/uracil phosphoribosyltransferase (FCU1) protein [194]. In co-administration with the non-cytotoxic 5-fluorocytosine (5-FC), TG6002 is able to catalyze the 5-FC conversion reaction into its cytotoxic forms, specifically killing infected tumor cells [195]. Recently, a Lister strain-derived oncolytic VV named GL-ONC, was used in phase I clinical trial, in nine patients with advanced peritoneal carcinomatosis. GL-ONC was administered intraperitoneally, every 4 weeks for up to four cycles at three different dose levels (10^7^–10^9^ PFU/mL). The results indicated that GL-ONC was well tolerated and did not show toxicities [196]. In another clinical trial, ACAM2000, a TK-positive VV strain, was used on twenty-four patients with solid tumors at stage III or IV. The dose range for ACAM2000 was between 1.4 × 10^6^ and 1.8 × 10^7^ PFU/mL, administered via intravenous, intra-tumoral and intra-peritoneal injections. This study demonstrated that ACAM2000 was very safe and in some patients led to a substantial tumor size reduction at six months post-treatment, or complete eradication of the solid tumor after three months to treatment [197].

### 2.8. Picornaviridae

*Picornaviridae* are viruses without an envelope and with an icosahedral capsid containing 32 capsomers. They replicate at the cytoplasmic level and consist of an RNA genome of about 8 kb that encodes four structural proteins. This family includes five genera, *Enterovirus, Rhinovirus, Cardiovirus, Aphtovirus,* and *Eparnavirus.* Rigvir belongs to the *Picornaviridae* family, genus *Enterovirus*, ECHO group (Enteric Cytopathogenic Human Orphan), type 7; it is not genetically modified, selected and adapted for the treatment of melanoma. Since 2011, Rigvir has been included in national guidelines for cutaneous melanoma in Latvia [198]. In 2015, Donina S. et al. [198] reported a clinical trial in which Rigivir has been administered intramuscularly with a minimum TCID50 dose of 10^6^ PFU/mL, and with a standard program that provides a total of 33 doses within three years. An improvement in survival and reduction in mortality was observed in patients with melanoma after treatment. Another research showed similar results in evaluating the effect of Rigivir on small cell lung cancer and histiocytic sarcoma [199]. In 2015, Zaurbek et al. [200] described a clinical case of advanced renal cell cancer that responded well to Rigvir treatment: tumor metastases are stabilized three months after the treatment. These promising results have reinforced the opinion that Rigvir could significantly prolong survival in patients without serious side effects and it could be recommended in long-term treatment. However, recently, Rigvir was suspended from the market in Latvia since the amount of the virus, which is supposed to destroy tumor cells, is of a much smaller amount than promised by the manufacturer.

## 3. OVs and HDACs as Combinatorial Therapy

HDACs are epigenetic modulators that act on the epigenetic asset of the cellular system [201,202,203,204,205,206,207,208].

HDACi are anticancer agents that induce cell cycle arrest, and, additionally, they can inhibit the growth and differentiation of cancer cells [204,205,209,210,211]. It is possible to distinguish HDACi in hydroxy acids (suberoylanilide hydroxamic acid, trichostatin A, or scriptaid), short-chain fatty acids (sodium butyrate or valproic acid), benzamide (MS-275 or entinostat), cyclic peptides (romidepsin or FR901228), and benzenesulfonamides (resminostat) [212]. Suberoylanilide hydroxamic acid (SAHA), also termed vorinostat, and trichostatin A (TSA) are FDA-approved pan-HDAC inhibitors. In detail, SAHA is a wide HDAC class I and II inhibitor known to block the growth of cancer cells, including cutaneous T-cell lymphoma, breast, and prostate cancer. It causes both transcriptional and non-transcriptional effects [213]. Regarding the former, different genes can be altered, reducing the activity of pro-growth and pro-survival proteins, such as Bcr-Abl, c-raf, and AKT [214]. In addition, SAHA can induce cell cycle arrest in G1 phase in cancer cells through the up-regulation of cyclin-dependent kinase inhibitor p 21 [215]. SAHA is also strongly related to apoptosis both by blocking Bcl-1 and Bcl-2 [216] and stimulating Bim, Bak, and Bax proteins [216,217,218]. TSA is a fungal antibiotic produced by Streptomyces hygroscopicus. It is structurally similar to SAHA and shows a broad spectrum of epigenetic activities by blocking HDACs class I and II too [219]. TSA represents a promising anticancer drug, specific for breast and prostate cancer [212]. Its mechanism of action includes the induction of cell cycle arrest and the expression of apoptosis-associated genes [220]. Scriptaid is a HDACi belonging to the hydroxamic class such as SAHA and TSA. Recent studies demonstrated the use of scriptaid in traumatic brain injury (TBI), where it acts by modulating the signaling pathway of protein kinase B (AKT) and promoting neuronal protection [221,222].

Sodium butyrate (NaB) is a class I and IIa HDACi. It has various functions both in terms of proliferation and differentiation, acting directly on the chromatin and facilitating the access of transcription proteins. It is characterized by a high anti-tumor capacity especially in prostate tumors and melanoma [212,221,223,224]. Another HDACi most used in oncolytic virotherapy is valproic acid (VPA). It is a broad class I and II HDACi and it represents the only FDA approved HDACi with antiproliferative action on both estrogen-sensitive and not-sensitive breast cancer cells [225]. VPA is also used in the treatment of melanoma and glioblastoma, by directly inhibiting HDACs and influencing both transcription-dependent and independent mechanisms [226,227,228]. Entinostat or MS-275 is a selective class I and IV HDACi. To date, it has not yet received approval for clinical use, but the US FDA allowed its combinatorial treatment with exemestane for the management of advanced breast cancer [229]. It is also used in the treatment of prostate carcinoma as it is capable of preventing the development of metastases by inducing cell death [221,230] and transcriptional activation of specific genes [231]. Romidepsin (FK228 or FR90128) is a depsipeptide belonging to the group of cyclic peptides, approved by the FDA in 2009 for the anticancer treatment of cutaneous T-cell lymphoma (CTCL) and in prostatitis carcinoma [232,233]. Romidepsin derives from the bacterium *Chromobacterium violaceum* and it acts by stopping the cell cycle and promoting apoptosis [212,234]. Resminostat, formerly named 4SC-201, is a FDA approved agent in the treatment of patients with HCC [235,236,237]. It inhibits class I and IIb HDAC by preventing the growth of cancer cells and enhancing apoptotic processes [238,239].

To date, several oncolytic viruses have been associated with HDACi with the aim of increasing antitumor efficacy and, on the other hand, reducing the antiviral response. HSV, AdV, reovirus, VSV, vaccinia virus (VV), paramyxoviruses, and parvovirus are the most representative.

### 3.1. Herpesviridae

Combinatorial therapy of G47Δ with TSA has been evaluated in various types of tumors such as glioma and colorectal cancer [138], indicating that it led to the inhibition of vascular endothelial growth factor (VEGF) and the degradation of Cyclin D1. Even in vivo, the antitumor and anti-angiogenesis efficacy have been confirmed in glioblastoma and colorectal carcinoma; in fact, an increase in survival and decrease in tumor growth has been observed in animals treated with the combinatorial treatment [240,241,242]. In addition to glioma, other studies described that TSA improved the anticancer activity of oHSV-1 in oral squamous cell carcinoma (SCC) increasing cytoplasmic nuclear factor-kappa B activity (NF-kB) [240]. By treating the cells with TSA and subsequently infecting them with R849, a variant of oHSV-1, there was an increase in viral production [138,240]. Treatment with TSA induced an increase in the acetylation levels of p65 and its translocation in the nucleus of cancer cells [240]. However, the effect of TSA decreased by using SN50, a NF-kB inhibitor, reducing the accumulation of p65 in the nucleus, and thus playing an important role in viral replication [241]. TSA can also up-regulate viral replication by increasing cytotoxicity [227,243]: it was able to upregulate the cyclin-dependent kinase inhibitor p21 and interrupt the cell cycle in the G0/G1 phase [138,240]. Another drug widely used in oncolytic virotherapy is VPA. In the study conducted by Jennings et al. [226], a JS-1 strain of HSV-1 encoding the GM-CSF was used resulting in effective activation of anti-melanoma immune response. Experiments have been conducted with both individual treatments (virus and VPA alone) and combinations, and it has been observed that the modified virus was able to produce type I interferon inducing the activation of immune response, but, with the addition of VPA, the anti-tumor immunity was enhanced. Many studies recurred to the use of VPA in the treatment of glioblastoma. In particular, it is reported that the time in which VPA is administered is essential. Otsuki et al. [228] indicated that HDAC inhibition was able to strengthen the anticancer potential of oncolytic virotherapy. They analyzed glioma cells co-treated and pre-treated with high doses of VPA (5-30 mM). Virus replication was calculated through the expression of Green Fluorescent Protein (GFP). In co-treatment assay, glioma cells were treated with oHSV and VPA at the same time, meanwhile, in pre-treatment test, cellular monolayer was stimulated before with HDACi and, later, it was infected with the virus. GFP intensity resulted higher in pre-treatment than in co-treatment assay. This effect has also been observed using other HDACi, such as Sodium Butyrate and TSA [228]. Furthermore, the study described above, has demonstrated that VPA played an important role in the innate antiviral response, as it blocked the type I interferon pathway (IFN I) during infection with rQNestin34.5 [228]. In glioma cells infected with oHSV-1, the expression of IFN I-sensitive genes, in particular STAT1, was evaluated, as it is known that INF I plays an important role in modulating antiviral responses. Transcription levels of IFN I-sensitive genes were assessed and a decrease in STAT1 levels was observed in the pre-treatment with VPA [228]. Moreover, an increase in viral yield occurred in STAT1 deficient cells. Therefore, VPA acts on STAT1 by improving viral yield. The effect of pre-treatment with VPA was also assessed in vivo using mice affected by glioma. The combinatorial treatment determined both an enhancement of viral propagation and higher survival compared to mice pre-treated with control solution (phosphate-buffered saline solution) [228]. In detail, in the study conducted by Ostuki et al., the brain tumor mice were pre-treated with VPA and PBS, and the next day they were infected with rQNestin34.5. Subsequently, the mice were killed and the levels of viral progeny present in both PBS and VPA treated mice were analyzed. The result indicated an increase in viral replication in animals pre-treated with valproic acid. In addition, survival studies were conducted on the same mice models. They were pre-treated with PBS and VPA and subsequently infected with oHSV-1. This treatment caused the death of animals pre-treated with PBS 20 days after infection, while, on the contrary, it contributed to the improvement of survival in mice pre-treated with VPA [228,241].

### 3.2. Adenoviridae

HDACi also acts on the transcription and translation of adenoviruses, so a combination of adenovirus and HDACi can represent a fair compromise [244]. This combination appears useful in cancer especially in esophageal carcinoma (ESCC) [245]. ESCC showed low levels of expression of the coxsackievirus and adenovirus (CAR) receptor. It has been demonstrated that TSA therapy induced an increase in CAR expression by promoting the oncolytic activity of adenovirus H101 [246]. Kitazono et al. evaluated the transgenic expression of adenovirus in cancer cells subjected to treatment with the HDACi romidepsin (FR901228). The authors described the treatment of malignant cells with FR901228 and the subsequent infection with Ad5 CMV-LacZ, a replication-defective type 5 adenovirus, devoid of the E1 and E3 gene. Pre-treatment caused an increase in the expression of CAR and integrin-alpha, important for mediating the attack of adenovirus on cells [247]. Effects of oncolytic virotherapy with AdV have also been observed in cervical cancer cells. In the study conducted by Han et al, the co-treatment of cancer cells with ZD55-TRAIL adenovirus and SAHA, resulted in a blockage of the cell cycle in the G2 phase and an increase of apoptosis [248]. ZD55-TRAIL is an engineered adenovirus that contains the ligand gene and promotes apoptosis associated with tumor necrosis factor (TRAIL) [248,249]. As evidence, in vivo studies have also shown that the combinatorial therapy (ZD55-TRAIL + SAHA) inhibited tumor growth, as SAHA functioned at the molecular level by preventing the up-regulation of p50 and p65 subunits of the nuclear factor-kB, which has been enhanced by ZD55- TRAIL [248].

### 3.3. Rhabdoviridae 

However, many cancer cells present residual innate activity that can generate resistance to viral propagation [138]. To overcome this limitation, HDACi combined with VSVΔM51 were used. In detail, oncolytic virotherapy has been combined with inhibitors such as SAHA and MS-275 in prostate cancer cells [138]. SAHA was able to regulate the expression of NF-kB target genes and block the expression of IFN, potentiating viral oncolysis, apoptosis, and NF-kB-mediated autophagy [250,251]. On the other hand, in vivo experiments have analyzed the combination SAHA or MS-275 and rVSV M Delta 51 in prostate, ovarian, and breast cancer xenograft models, and showed enhanced survival [252,253]. Furthermore, Muscolini et al identified SIRT1 as a probable factor limiting viral infection in prostate cancer cells. SIRT 1 is a class III HDACi, also called NAD-dependent sirtuin 1. Different studies showed that SIRT 1 played an important role both in neurodegenerative pathologies and also in carcinomas [254]. Indeed, it has been considered a restriction factor known for its importance in prostate cancer, where it acted on the permissiveness of specific tumor cells. SIRT1 interfered with the prostate cancer cells making them permissive to the rVSV M Delta 51 infection [255]. 

### 3.4. Reoviridae 

Combinatorial therapy between HDACi and oncolytic reoviruses has also been evaluated in patients with multiple myeloma (MM). It is an incurable hematologic neoplasm, caused by the neoplastic transformation of a cell of the B lymphocyte line. Life expectancy is reduced: in most cases, death occurs within 5 years of diagnosis, and, in cases where the tumor is aggressive, within 24 months [256,257,258]. In a study conducted in 2016, the combination of several HDACi and Reolysin demonstrated to increase the therapeutic potential in patients with MM [258]. By performing Western blot and flow cytometry analysis, lower expression of the reovirus receptor junctional adhesion molecule 1 (JAM-1) was observed in resistant tumor cells, infected with different amounts of virus, compared to sensitive ones. Furthermore, it has been seen that the expression of JAM-1 can be epigenetically regulated: a dose-dependent increase has been observed by treating the cells with various HDACi, including SAHA and MS-275. In particular, MS-275 was able to up-regulate the expression of the JAM-1 protein and the combination with Reolysin increased antiviral activity by killing not only MM cells but also Mantle cell lymphoma (MLC). In addition, Jaime-Ramirez et al. assessed the impact of the combination of oncolytic reovirus and SAHA in head and neck squamous cell carcinomas (HNSCC), demonstrating an improvement in viral replication and immune-mediated anti-cancer responses both in vitro and in vivo [259].

### 3.5. Parvoviridae 

Important results have been obtained in cervical cancer and pancreatic duct adenocarcinoma by the combination of HDACi. In detail, rat parvovirus H1PV has oncolytic and suppressive activity against the tumor. It has been reported that co-treating cancer cells with VPA and H1PV, as a result the onset of oxidative stress and apoptosis of cancer cells occurred [260]. The same effects have been observed by using H1PV and NaB at sub-lethal doses. There was an increase in viral oncotoxicity determining the eradication of neoplasm, but, on the other hand, there was the regression of carcinoma [138,185].

### 3.6. Paramyxoviridae 

It has been shown that under optimal conditions, when cells were infected with the P/V-CPI mutant alone, it caused an increase in the production of IFN beta, while in cells infected with the oncolytic mutant virus and treated with scriptaid, there was a reduction in the production of INF and an increase in viral propagation in cancer cells [192]. Significant progress has also been assessed in the treatment of HCC in which the oncolytic measles vaccine virus (MeV) has been associated with the oral HDACi resminostat (Res) [238]. Res-MeV co-treatment increased viral replication and apoptosis, and improved primary infections. Furthermore, Res could exert a remarkable effect on innate cellular immunity, as it could prevent the activation of genes stimulated by IFN [238].

### 3.7. Poxviridae

Currently, VV is under study and their activity can be enhanced by the use of HDACi [252,261]. Among the various HDACi, TSA represents the VV enhancer both in vitro and in vivo. Indeed, TSA caused a greater effect in vitro than other inhibitors, enhancing viral replication and the killing of infection-resistant tumor cells and, on the contrary, it was able to reduce toxicity to the mice [227]. Even in vivo studies with human colon carcinoma xenografts have shown that the combinatorial treatment resulted in improved survival [138,262].

## 4. OVs and DNMTi as Combinatorial Therapy

DNMTs are coding enzymes that play an important role in epigenetic regulation. Mammals encode six DNMTs: DNMT1, DNMT3A, and DNMT3B are cytosine-5 DNMTs, meanwhile, DNMT2 and DNMT3L are not canonical demethylating enzymes, as they do not contain the catalytic activity [263]. Recently, DNMT3C has been identified as a new methyltransferase enzyme involved in mouse development and fertility [264]. DNMT1 acts to maintain the methylation status [265], and, on the contrary, DNMT3A and DNMT3B participate in the de novo methylation [266]. DNMT2 does not methylate genomic DNA but the anticodon loop of aspartic acid transfer RNA [267]. Lastly, DNMT3L does not possess any catalytic domain, but it can stimulate DNMT3A and DNMT3B activity [268]. They perform different functions by acting in particular on the remodeling of chromatin and they are responsible for the up/down expression of proteins causing the onset of different pathologies [269]. Aberrant DNA methylation is a phenomenon widely observed in many cancer types, such as colon, breast, liver, bladder, ovarian, esophageal, prostate, and bone cancers [270,271,272,273,274]. Furthermore, the role of DNA methylation in common human pathologies has also been investigated, in particular in neurological disorders [275,276] and autoimmune diseases [277,278,279]. It has been largely reported that DNA hypermethylation is strongly related to carcinogenesis. In this scenario, demethylating drugs could represent promising anticancer agents. Among DNMTi, azacitidine (5-AZA) and decitabine (5-aza-2ʹ-deoxycytidine) have been approved by the FDA for the treatment of acute myeloid leukemia and myelodysplastic syndrome. These are cytidine analogues that need to be incorporated into the genome during the S phase of the cell cycle to perform their action. While 5-AZA can be integrated into both RNA and DNA, decitabine is incorporated only into DNA [269]. Similarly to HDACi, the combinatorial therapy of DNMTs with oncolytic viruses could lead to the improvement of tumor destruction together with the stimulation of the immune system (Table 2).

### 4.1. Herpesviridae

Okemoto et al have demonstrated that by combining 5-aza with the oncolytic HSV-1 rQNestin34.5, remarkable results were obtained both in vivo and in vitro in the treatment of glioma [280]. ICP34.5 gene promoter has been shown to contain an island rich in CpG and, as a result, it can be demethylated by using specific demethylating agents. By treating glioma cells with oHSV and 5-aza, there was an increase in the viral replication, as reported by the high expression of some viral genes and by the increase in the number and size of infected GFP-positive glioma cells [280]. Furthermore, Okemoto et al demonstrated that rQNestin34.5 and 5-aza can act synergistically causing apoptosis of glioma tumor cells. Interesting results have been observed also in vivo, with an improvement in survival in mice bearing orthotopic human gliomas [280]. To improve anticancer responses, combinatorial experiments have also been performed with 5-aza and the bovine herpes virus type 1 (BHV-1) [228,280,281]. BHV-1 has a replication cycle very similar to that of HSV-1 and it is responsible for the onset of respiratory infections [281,283]. Treatment with DNMTi increases oncolytic replication both in vivo and in vitro. Monotherapy and combinatorial experiments were conducted in vitro, using cells derived from spontaneous breast fibrosarcomas (LCRT). A single treatment with 5-aza enhanced viral replication, meanwhile the combination of the two agents resulted in an apoptotic increase in cancer cells [281]. Instead in vivo studies have been conducted on cotton mice treated with BHV-1 and 5-aza. It has been shown that the use of OV alone caused a delay in tumor growth, whereas the combinatorial treatment decreased the production of secondary lesions [280].

### 4.2. Adenoviridae

Chen et al evaluated a new therapeutic approach for the treatment of tumors, which cannot be included in DNMT inhibition but it reflects similar purposes. In detail, by using oncolytic adenoviruses and the RNA interference (RNAi) of the enzyme O 6-methylguanine DNA methyltransferase (MGMT), there was a potentiation of the antitumor activity of the drug temozolomide (TMZ) [284]. TMZ is mainly used for the treatment of malignant melanoma and glioma [284,285,286]; however, like the other drugs, prolonged use can induce resistance by producing the O 6- methylguanine mutagen and causing DNA damage. Such damage can be repaired by MGMT [284,287]. It has been observed that inhibition of MGMT improved the antitumor activity of the drug [284]. The combination between oncolytic adenoviruses and shRNA targeting MGMT activity could be an effective approach for fighting resistance to TMZ and for improving anticancer outcomes.

### 4.3. Rhabdoviridae

Therapeutic studies have also been conducted to enhance the treatment of onco-hematological diseases such as acute T-cell lymphocytic leukemia. It has been shown that the single use of OV and/or decitabine reduced the antitumor efficacy, meanwhile, on the contrary, the combination of the two agents led to several significant advantages [288]. Hastie et al used murine EL-4 cells from acute T-cell lymphocytic leukemia. Pre-treatment with the oncolytic virus VSVΔM51 before therapy with the DNMTi, caused tumor remission in 70% cases [289]. Cells which survived two consecutive treatments with the epigenetic modulator were more sensitive to oncolytic viral therapy, leading to durable remissions.

## 5. OVs and miRNA: Promising Combinatorial Treatment

In the past decade, the study of miRNAs has largely influenced the field of oncolytic virotherapy. Specific miRNAs target sequences can be integrated into the viral genome and can regulate viral proteins, improving the safety profile and strengthening the anticancer efficacy of oncolytic viruses (Table 3).

miRNAs are small non-coding RNA molecules approximately 22 nucleotides long that can negatively regulate gene expression at the post-transcriptional level [305]. The miRNAs participate in numerous cellular functions including proliferation, apoptosis, metabolism, and it is not surprising that their dysregulation may be involved in tumorigenesis phenomena [306,307,308,309]. In order to make OV tropism more selective in cancer cells and reduce the toxicity, the downregulation of specific miRNAs has been used in oncolytic virotherapy. In this scenario, synthetic target sequences complementary to specific miRNAs have been inserted in the UTRs of viral genes essential for replication. This approach promotes the degradation of the viral genome in healthy tissues, but not in cancer cells [131,310,311,312]. This targeting strategy depends on the small size of miRNAs that can be inserted into the viral genome without compromising normal replication in tumor tissues. Its usefulness has been widely demonstrated and tissue specificity has been improved for many oncolytic viruses [304,311,313,314,315,316].

### 5.1. Herpesviridae

The first study on oncolytic HSV and miRNA was that of Anesti et al. They reported an innovative strategy in which miRNAs mediated gene silencing [317]. Another approach was developed by Li et al to fight NSCLC. They described the effects of miRNA-145 regulated HSV-1 [318,319] whose expression was low in NSCLC tumor cells. A complementary target sequence to miRNA-145 was introduced into the 3’ UTR of the ICP27 gene, which encodes a glycoprotein essential for HSV attachment and fusion to the host cell. The results demonstrated that ICP27 protein level was higher in tumor cells than in healthy cells, indicating that this type of regulation could control HSV-1 by selectively killing NSCLC cells in vitro [292]. Generally, miRNA-21 was found to be upregulated in cancer cells [320]. An inverse miRNA control setup was created, in which miR-21 was used in cancer cells to induce, rather than repress, HSV replication. This study has shown that a viral gene under the control of miR-21 limited viral replication in healthy cells, where miR-21 was downregulated, and, at the same time, it induced a vigorous replication in cancer cells expressing miR-21 [290]. Many studies have used oncolytic adenovirus in combination with hepatic specific miRNAs, such as miR-122, to counteract hepatotoxicity and increase the virus specificity.

### 5.2. Adenoviridae

A first study was performed by Ylosmaki et al, which inserted the EA1 gene under the control of miR-122 in serotype 5 adenovirus (Ad5) [321]. In this case, six copies of the target sequence for miR-122 were used to prevent the replication of Ad5 in healthy liver tissue. Differently from the wild-type Ad5, the engineered virus did not produce an increase in serum liver enzyme levels in infected mice. These results have prompted other studies that have combined miR-122 with miR-19, also specific for hepatocytes and downregulated in cancer cells. Both miRNAs were inserted into the 3′ UTR region of EA1 gene controlled by hTERT promoter, obtaining an increase in OV adenovirus specificity [322]. In another study, eight target sequences that recognized the members of the miR-148a/miR-152 family, were inserted downstream of the E1A gene. This modification effectively inhibited adenoviral infection in healthy pancreatic tissue and, on the contrary, it has improved the viral anti-tumor activity in pancreatic tumors [323]. Other genes essential to adenovirus replication have been regulated by miRNA target sequences, such as the gene encoding L5 protein: eight binding sequences for miR-148a have been added downstream. The mice treated with Ad-L5-8miR148aT showed reduced adenovirus-induced hepatotoxicity and full lytic activity in tumor cells [295]. Therefore, the control of the late proteins through miRNAs is also a strategy for improving viral selectivity. The presence of viral proteins in normal tissues could create immunogenic reactions, as well as inflammation and cell death. It represents an undesirable effect, which thanks to the intervention of miRNAs can be shot down in normal cells, increasing the safety profile and therapeutic index in oncolytic virotherapy. Furthermore, the ability to introduce multiple miRNA elements into the same viral genome can simultaneously eliminate various off-target toxicities, and thus it could eliminate the toxicity coming from a systemic infection. Finally, by selecting miRNAs with high levels of expression and incorporating multiple copies into the viral genome, saturation phenomena or point mutations during infection can be avoided. However, other studies and clinical trials will need to be performed before the therapeutic potential of this innovative approach and its safety can be assessed in humans.

### 5.3. Picornaviridae

One of the first miRNA-regulated oncolytic viruses was the Coxsackievirus B3 (CVB3) characterized by the strong ability to lyse human cells of NSCLC [324,325]. Target sequences for miR-34a have been inserted both in the 3′ UTR and 5′ UTR of the viral genome. The recombinant virus, called 53a-CVB, showed minimal levels of toxicity in healthy tissues and, furthermore, retained its full oncolytic activity in xenotransplant mice with human lung cancer [303]. 53a-CVB virus represents the first OV regulated by miR-34a and an innovative springboard for the development of safe and effective anti-cancer therapies. In addition, the Coxsackievirus A21 (CVA21) was modified by Kelly et al. [311] inserting target sequences complementary to miR-206 and miR-133a, specific to skeletal muscle tissue. A21 wild-type virus causes lethal myositis in tumor bearing mice. On the contrary, the recombinant virus retained its replication ability in cancer cells, causing total regression of subcutaneous tumors, and did not replicate in healthy cells expressing complementary miRNAs, thereby reducing myotoxicity, and retaining the oncolytic potential [311].

Another member of the Picornaviridae family, Mengovirus, can cause encephalitis and myocarditis in multiple mammalian species. It has shown oncolytic activity but its use can also cause side effects. In order to improve the safety profile and reduce toxicity, Ruiz et al. engineered the virus by inserting target sequences complementary to miR-124 (enriched in nerve tissue) in the 5′ UTR of the viral genome, and sequences complementary to miR-133 and miR-208 (enriched in heart tissue) were introduced in the 3′ UTR [304]. In vivo toxicity assays confirmed that miR-124, inserted within the 5′ UTR of the viral genome, suppressed viral replication in the central nervous system, while miR-133 and miR-208 inhibited viral replication in the heart tissue. The recombinant virus was called VMC24-NC and retains a full oncolytic power if administered intratumor or intravenously [304]. This study has shown that the simultaneous use of multiple targets for miRNA reduces the saturation potential of a single miRNA.

## 6. Conclusions

**Figure 1:**
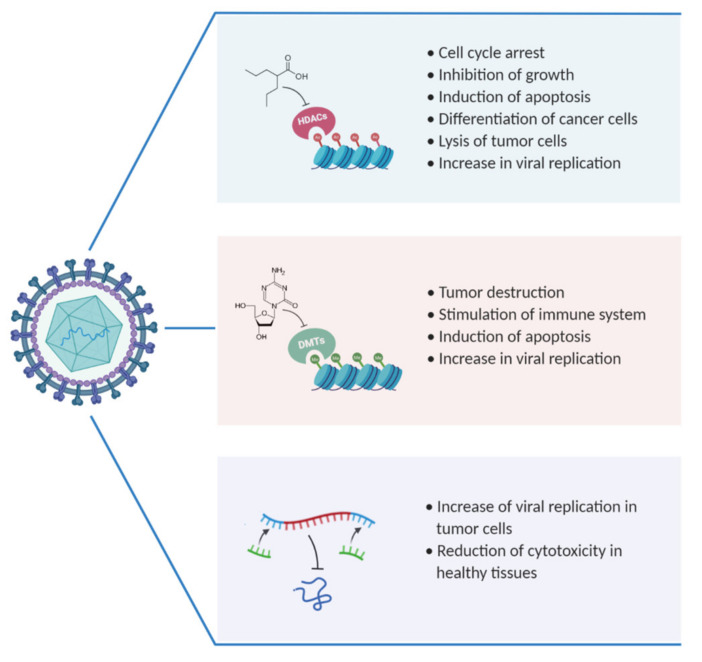
Summarizes the effects of combinatorial treatment by the use of OVs, epigenetic modulators and miRNA. Created with BioRender.com.

At the present, much evidence demonstrates that tumor cells are deregulated by epigenetic mechanisms, apart from genetic alterations. On the other hand, OVs are promising therapeutic agents which are developing in the cancer treatment field. They are great biotherapeutic platforms to be combined with epigenetic drugs, able to cut down cellular antiviral response, promote the destruction of cancer cells and potentiate the immune response (Figure 1). Although in many clinical trials, no mortality following virotherapy has been reported, except sporadically, the advancement of treatments in combination with other molecules, and the use of different oncolytic viruses, still raises safety concerns. Although genetic manipulation has improved the tropism of oncolytic viruses for cancer cells, it is possible to recur to genetic recombination or mutation with the consequent production of unexpected toxic effects. However, many studies report that the effects of virotherapy are generally well-tolerated and that the most common side effects, when present, are flu-like. One of the main mechanisms of resistance to virotherapy is represented by innate immunity. Through the production of neutralizing antibodies, the immune system can limit the ability of the virus to replicate sufficiently to spread into neighboring tumor tissues. In this case, the use of immunosuppressant drugs in the early stages of treatment could resolve the problem [326]. Unfortunately, this option could exclude immunocompromised patients or people with active viral infections. In the last decade, studies on the development and improvement of OVs at the preclinical level have increased. Furthermore, new strategies adopted, such as the introduction of epigenetic modulators in virotherapy, are bringing more safety and selectivity to cancer cells, increasingly reducing collateral damage to healthy cells [327]. However, an important feature reported by several studies is represented by viral pharmacodynamics and pharmacokinetics, which allows constant monitoring of the immune response and viral load, both essential for understanding the course of treatment and suggesting changes in the case of problems. Moreover, apart from combining OVs with other molecules, it should be very interesting to combine virotherapy with standard cancer therapeutics like chemotherapy and radiotherapy, with the aim to improve time survival in advanced cancer patients. For example, in a Phase I trial by Mell et al. [328] is GL-ONC, a modified oncolytic VV combined with cisplatin and radiotherapy in patients with advanced head and neck cancers which has shown to improve 1-year progress-free survival. In this scenario, the prospects for the future will be to improve combinations and OVs, increase efficiency, safety, and treatment times, with particular attention to those tumors that are difficult to eradicate and resistant to chemotherapy.

Additional in vitro and in vivo studies are needed in order to extend the panel of OVs and epigenetic modulators used to reach the clinic in the near future, to further improve their therapeutic impact.

## Figures and Tables

**Table 1:** Oncolytic viruses subjected to clinical trials.

Virus Family	Virus	Institution	Tumor	Phase	Status	Trial N°	Source
*Herpesviridae*	Imlygic	BioVex Limited	Melanoma	III	Completed	NCT00769704 NCT01368276	[67,68]
		BioVex Limited	Pancreatic Cancer	I	Completed	NCT00402025	[69]
		BioVex Limited	Squamous Cell Carcinoma; Head and Neck\Cancer	III	Completed	NCT01161498	[70]
	G47∆	The University of Tokyo Hospital	Prostate cancer	I	Completed	UMIN000010463	[32]
		The IMSUT Hospital	Glioblastoma	II	Completed	UMIN000015995	[71]
	G47∆	The University of Tokyo Hospital	Prostate cancer	I	Completed	UMIN000010463	[32]
		The IMSUT Hospital	Glioblastoma	II	Completed	UMIN000015995	[71]
	rQNestin34.5	National Institutes of Health (NIH); Candel Therapeutics, Inc.; Dana-Farber Cancer Institute	Glioma; Astrocytoma; Glioblastoma;	I	Recruiting	NCT03152318	[72]
	G207	MediGene	Glioma; Astrocytoma; Glioblastoma;	I, II	Completed	NCT00028158	[73]
		MediGene	Malignant glioma	I	Completed	NCT00157703	[74]
*Adenoviridae*	H101 (Oncorine)	Sun Yat-sen University	Hepatocellular Carcinoma	III	Recruiting	NCT03780049	[75]
		Fudan University	Malignant Ascites	II	Recruiting	NCT04771676	[76]
	DNX-2440	DNAtrix, Inc., H. Lee Moffitt Cancer Center and Research Institute	Colon cancer; Colorectal Cancer; Breast Cancer; Melanoma; Renal Cell Cancer; Sarcoma; Squamous Cell Carcinoma	I	Recruiting	NCT04714983	[77]
		Clinica Universidad de Navarra, Universidad de Navarra, DNAtrix, Inc.	Glioblastoma	I	Recruiting	NCT03714334	[78]
	DNX-2401 (Delta-24-RGD)	Clinica Universidad de Navarra, Universidad de Navarra, DNAtrix, Inc.	Glioblastoma	I	Completed	NCT01956734 NCT02197169 NCT01582516	[79,80,81]
	TILT-123	TILT Biotherapeutics Ltd.	Solid Tumor; Metastatic Melanoma	I	Recruiting	NCT04695327 NCT04217473	[82,83]
	VCN-01	VCN Biosciences, S.L.	Solid Tumors; Pancreatic Adenocarcinoma	I	Completed	NCT02045602 NCT02045589	[84,85]
		Institut Català d’Oncologia, VCN Biosciences, S.L. BioClever 2005 S.L. AstraZeneca	Head and Neck Neoplasms Carcinoma	I	Recruiting	NCT03799744	[86]
	LOAd703	Lokon Pharma AB	Pancreatic Cancer	I, II	Recruiting	NCT02705196	[87]
		Lokon Pharma AB, Precision Oncology LLC	Malignant Melanoma	I, II	Recruiting	NCT04123470	[88]
		Lokon Pharma AB, Uppsala University	Pancreatic Adenocarcinoma; Ovarian Cancer; Biliary Carcinoma; Colorectal Cancer	I, II	Recruiting	NCT03225989	[89]
	ICOVIR-5	Institut Català d’Oncologia	Melanoma	I	Completed	NCT01864759	[90]
	CG0070	CG Oncology, Inc., Merck Sharp & Dohme Corp.	Non Muscle Invasive Bladder Cancer	II, III	Recruiting	NCT04387461 NCT04452591 NCT02365818	[91,92,93]
	Enadenotucirev	University of Oxford, PsiOxus Therapeutics Ltd. Cancer Research UK	Rectal Cancer	I	Recruiting	NCT03916510	[94]
	CAdVEC	Baylor College of Medicine, The Methodist Hospital System, Daniel Wang, Baylor College of Medicine	Bladder Cancer; Head and Neck Squamous Cell Carcinoma; Breast cancer; Colorectal Cancer; Pancreatic Cancer	I	Recruiting	NCT03740256	[95]
	ORCA-010	Orca Therapeutics B.V., CMX Research	Prostate Cancer	I, II	Recruiting	NCT04097002	[96]
	NG-641	PsiOxus Therapeutics Ltd.	Epithelial Tumor	I	Recruiting	NCT04053283	[97]
*Reoviridae*	Reolysin	Oncolytics Biotech	Malignant Glioma	I	Completed	NCT00528684	[98]
		Oncolytics Biotech	Pancreatic Adenocarcinoma	I	Completed	NCT02620423	[99]
		Emory University, Bristol-Myers Squibb Oncolytics Biotech University of Utah City of Hope Medical Center Phylogeny	Recurrent Plasma Cell Myeloma	I	Recruiting	NCT03605719	[100]
		Oncolytics Biotech	Squamous Cell Carcinoma of the Lung	II	Completed	NCT00998192	[101]
		Oncolytics Biotech	Squamous Cell Carcinoma of the Head and Neck	II	Completed	NCT00753038	[102]
		Oncolytics Biotech	Melanoma	II	Completed	NCT00984464	[103]
		Incyte Corporation; Oncolytics Biotech; National Cancer Institute (NCI)	Breast Cancer	II	Recruiting	NCT04445844	[104]
		Oncolytics Biotech	Carcinoma, Squamous Cell of the Head and Neck	III	Completed	NCT01166542	[105]
		National Cancer Institute (NCI)	Ovarian Cancer	II	Completed	NCT01199263	[106]
*Parvoviridae*	H1-PV	Oryx GmbH & Co. KG	Glioblastoma Multiforme	I, II	Completed	NCT01301430	[107,108]
		Oryx GmbH & Co. KG	Pancreatic Ductal Carcinoma	I, II	Completed	NCT02653313	[109]
*Paramyxoviridae*	*Measles virus*	National Cancer Institute (NCI)	Anaplastic Astrocytoma; Anaplastic Oligodendroglioma Mixed Glioma; Recurrent Glioblastoma	I	Completed	NCT00390299	[110]
		National Cancer Institute (NCI)	Ovarian Cancer; Primary Peritoneal Cavity Cancer	I	Completed	NCT00408590	[111]
		University of Arkansas	Multiple Myeloma	II	Completed	NCT02192775	[112]
		National Cancer Institute (NCI)	Breast Cancer	I	Recruiting	NCT04521764	[113]
		Mayo Clinic, National Cancer Institute (NCI)	Ovarian Carcinoma, Peritoneal Carcinoma, Fallopian Tube Transitional Cell Carcinoma	I, II	Recruiting	NCT02068794	[114]
		Mayo Clinic, National Cancer Institute (NCI)	Malignant Mesothelioma	I	Completed	NCT01503177	[115]
		Mayo Clinic, National Cancer Institute (NCI)	Plasma Cell Myeloma	I, II	Completed	NCT00450814	[116]
		Mayo Clinic, National Cancer Institute (NCI)	Malignant peripheral nerve sheath tumor	I	Recruiting	NCT02700230	[117]
*Poxviridae*	*Vaccinia virus*	Assistance Publique-Hopitaux de Paris	Glioblastoma; Brain Cancer;	I, II	Recruiting	NCT03294486	[118]
		Jennerex Biotherapeutics, SillaJen, Inc.	Hepatocellular Carcinoma	II, III	Completed	NCT00554372 NCT01636284	[119,120]
		Jennerex Biotherapeutics	Melanoma	I, II	Completed	NCT00429312	[121]
		SillaJen, Inc., Regeneron Pharmaceuticals	Renal Cell Carcinoma	I, II	Recruiting	NCT03294083	[122]
		Jennerex Biotherapeutics, Green Cross Corporation	Hepatic carcinoma	I	Completed	NCT00629759	[123]

OV and its manufacturing company are reported in the table. Furthermore, the type of tumor and the progress of clinical trials with the relative code are indicated as reported on https://clinicaltrials.gov/, accessed on 31 May 2021. The detailed description of OVs genome structure and their use in clinics are reported throughout the text.

**Table 2:** Oncolytic virotherapy in combination with epigenetic modulators used in cancer therapy. It is indicated OV and epigenetic drug. In addition, the tumor type and the effect of the combinatorial treatment are reported.

Virus Family	Oncolytic Virus	Epigenetic Modulators	Tumor Type	Effects	Source
*Herpesviridae*	G∆47	TSA	Glioma; Colorectal cancer	Reduced VEGF and cyclin D1	[138]
	R849	TSA	Oral squamous cell carcinoma (SCC)	Increased NF-kB activity Increased viral production Increased p21 interrupt cell cycle in G0/G1 phase	[240]
	rQNestin34.5	VPA; TSA; NaB 5-AZA	Glioblastoma Glioma	Increased viral replication Increased IFN-I expression Increased tumor apoptosis	[228,280]
	BHV-1	5-AZA	Spontaneous breast fibrosarcomas	Increased tumor apoptosis	[281]
	H101	TSA; romidepsin	Esophageal squamous cell carcinoma (ESCC)	Increased CAR and viral activity Increased viral entry	[247]
*Adenoviridae*	ZD55-Trial	SAHA	Cervical cancer	Increased cell cycle block in phase G2 Increased apoptosis	[248,249]
*Rhabdoviridae*	VSV∆51	vorinostat; MS275; SIRT1; decitabine	Prostate cancer T-cell lymphocytic leukemia	Increased apoptosis Increased viral oncolysis Increased remission tumor	[138,176,255]
*Reoviridae*	RV	entinostat	Multiple myeloma (MM); Head and neck squamous cell carcinomas (HNSCC)	Increased antiviral activity	[259,282]
*Parvoviridae*	H1PV	VPA; NaB	Cervical cancer; Pancreatic cancer Carcinoma	Increased apoptosis Increased viral oncotoxicity	[138,185]
	P/V-CPI	scriptaid	Small cell lung cancer; Laryngeal carcinoma cells	Increased apoptosis	[192]
*Paramyxoviridae*	MeV	Res	HCC	Increased apoptosis Increased viral replication	[238]
*Poxviridae*	VV	TSA	Colon carcinoma	Increased viral replication	[138,262]

**Table 3:** miRNA-regulated vectors used in virotherapy. Engineered virus, tumor cells targeted by combinatorial treatment and the associated effects are reported.

Virus Family	Engineered Virus	miRNA	Target cell/Tissue	Results/Effects	Source
*Herpesviridae*	dnU L 9-T21	miR-21	Glioblastoma cell lines	Increased cell specificity	[290]
	KG4: T-124	miR-124	Brain tissue	Increased safety	[291]
	AP27i145	miR-145	Non-small cell lung cancer cells	Increased selectivity	[292]
	LCSOV	miR-7;miR-122; miR-124	HCC	Increased selectivity Increased safety	[293]
*Adenoviridae*	AdΔ24.CMV-GFP	miR-26b	Prostate cancer	Increased propagation Reduced spread	[294]
	Ad-L5-8miR148aT	miR-148a	Hepatocytes/liver	Increased hepatotoxicity Increased anticancer response	[295]
	ICOVIR15	miR-99b; miR-485	Pancreatic Cancer	Increased anticancer activity Increased oncolytic potential	[296]
	AdCN205-IL-24-miR-34a	miR-34a	HCC	Complete tumor regression	[297]
	rAd-199T-miR-221	mir-221	HCC	Reduced apoptosis Increased miR-221 levels	[298]
	Ad-199T	miR-199	HCC	Reduced hepatotoxicity Increased cell specificity	[299]
*Paramyxoviridae*	MV-EGFP-H miRTS7, MV-EGFP-H miRTS7rc, MV-EGFP-H miRTS122, MV-EGFP-H miRTS122rc, MV-EGFP-H miRTS124	miR-124; miR-125b; miR-7	Brain tissue	Increased replicative control	[300]
	SLAM of Measles Virus	miR-31 miR-128	Glioblastoma	Increased infectivity Increased anticancer effect	[301]
*Poxviridae*	VV-miR-34a VV-Smac	miR-34a	Multiple Myeloma	Increased anticancer effect Increased apoptosis Reduced tumor growth	[302]
*Picornaviridae*	53a-CVB	miR-34a/c	Human non-small cell Lung cancer cells	Reduced cytotoxicity	[303]
	vMC24-NC	miR-124 miR-125 miR-133 miR-208 miR-142	Nervous tissue Cardiac tissue Hematopoietic tissues	Reduced pathogenesis Increased safety	[304]
